# Proteomic signatures of eosinophilic and neutrophilic asthma from serum and sputum

**DOI:** 10.1016/j.ebiom.2023.104936

**Published:** 2023-12-20

**Authors:** Khezia Asamoah, Kian Fan Chung, Nazanin Zounemat Kermani, Barbara Bodinier, Sven-Erik Dahlen, Ratko Djukanovic, Pankaj K. Bhavsar, Ian M. Adcock, Dragana Vuckovic, Marc Chadeau-Hyam, the U-BIOPRED Study Group

**Affiliations:** a MRC Centre for Environment and Health & Department of Epidemiology and Biostatistics, Faculty of Medicine, School of Public Health, Imperial College London, United Kingdom; b Data Science Institute, Department of Computing, Imperial College London, United Kingdom; c National Heart and Lung Institute, Imperial College London, London, United Kingdom; d Royal Brompton and Harefield Hospital, London, United Kingdom; e Institute of Environmental Medicine and Department of Medicine Huddinge, Karolinska Institutet, Stockholm, Sweden; f Department of Respiratory Medicine, Karolinska University Hospital, Stockholm, Sweden; g Clinical and Experimental Sciences, Faculty of Medicine, University of Southampton, Southampton, United Kingdom; h National Institute for Health Research Southampton Biomedical Research Centre, Southampton, United Kingdom

**Keywords:** Proteomics, LASSO, Sputum, Serum, Eosinophil, Neutrophil, Asthma

## Abstract

**Background:**

Eosinophilic and neutrophilic asthma defined by high levels of blood and sputum eosinophils and neutrophils exemplifies the inflammatory heterogeneity of asthma, particularly severe asthma. We analysed the serum and sputum proteome to identify biomarkers jointly associated with these different phenotypes.

**Methods:**

Proteomic profiles (N = 1129 proteins) were assayed in sputum (n = 182) and serum (n = 574) from two cohorts (U-BIOPRED and ADEPT) of mild-moderate and severe asthma by SOMAscan. Using least absolute shrinkage and selection operator (LASSO)-penalised logistic regression in a stability selection framework, we sought sparse sets of proteins associated with either eosinophilic or neutrophilic asthma with and without adjustment for established clinical factors including oral corticosteroid use and forced expiratory volume.

**Findings:**

We identified 13 serum proteins associated with eosinophilic asthma, including 7 (PAPP-A, TARC/CCL17, ALT/GPT, IgE, CCL28, CO8A1, and IL5-Rα) that were stably selected while adjusting for clinical factors yielding an AUC of 0.84 (95% CI: 0.83–0.84) compared to 0.62 (95% CI: 0.61–0.63) for clinical factors only. Sputum protein analysis selected only PAPP-A (AUC = 0.81 [95% CI: 0.80–0.81]). 12 serum proteins were associated with neutrophilic asthma, of which 5 (MMP-9, EDAR, GIIE/PLA2G2E, IL-1-R4/IL1RL1, and Elafin) complemented clinical factors increasing the AUC from 0.63 (95% CI: 0.58–0.67) for the model with clinical factors only to 0.89 (95% CI: 0.89–0.90). Our model did not select any sputum proteins associated with neutrophilic status.

**Interpretation:**

Targeted serum proteomic profiles are a non-invasive and scalable approach for subtyping of neutrophilic and eosinophilic asthma and for future functional understanding of these phenotypes.

**Funding:**

U-BIOPRED has received funding from the 10.13039/501100010767Innovative Medicines Initiative (IMI) Joint Undertaking under grant agreement no. 115010, resources of which are composed of financial contributions from the European Union's Seventh Framework Programme (FP7/2007–2013), and 10.13039/100013322European Federation of Pharmaceutical Industries and Associations (EFPIA) companies' in-kind contributions (www.imi.europa.eu). ADEPT was funded by 10.13039/100004331Johnson & Johnson/Janssen pharmaceutical Company.

**Research in context**

**Evidence before this study**

To identify relevant studies for this analysis, we searched PubMed and Google Scholar using search terms that investigated identifying proteomic biomarkers for “eosinophilic asthma”, “neutrophilic asthma” as well as proteomic biomarkers linked to “blood eosinophil counts” and “blood neutrophil counts” in relation to asthma. The search was limited to studies looking into identifying biomarkers from assays from conception to date.

The literature search identified studies focusing on different asthma phenotypes; however, studies usually had a smaller subset of analytes (e.g. around 90) and sometimes even smaller populations (e.g. 30–200). Furthermore, there are some known biomarkers for eosinophilic and neutrophilic asthma linked to the types of inflammation that can potentially govern these phenotypes e.g. IL-5, IgE, and IL-8 respectively.

However, studies that have previously used larger assays tended to focus on one study population instead of a combination. In comparison to what we have done in this study, there has not been a comparison of biomarkers in multiple media for different asthma phenotypes with a large biomarker assay as we are proposing.

**Added value of this study**

Of the 1129 assayed proteins, we found that the serum levels of 13 and 12 of them were jointly explanatory of eosinophilic and neutrophilic status, with an AUC of 0.83 (95% CI 0.82–0.83) and 0.91 (95% CI 0.90–0.92), respectively. These explained at least as much as the selected sputum proteins and most of them (i) were complementary to established clinical factors, and (ii) were robust to the definition of eosinophilic and neutrophilic status. The 13 serum proteins associated with eosinophilic asthma status included PAPP-A, TARC/CCL17, CCL28, CO8A1, IgE, IL-5-Rα and pointed towards cell growth, differentiation, and proliferation pathways as well as T helper 2 (Th2) immune response and possibly integrin-mediated and extracellular matrix signalling. Of the serum proteins associated with neutrophilic asthma status, five (MMP 9, EDAR, PLA2G2E, IL1RL1, and Elafin) were complementary to established clinical factors and related to airway remodelling pathways in asthma cases (MMP-9), neutrophil recruitment, with one possible anti-inflammatory protein, Elafin. These associations were replicated in repeated measurements in our study population and were robust to the clinical definition of these asthma subtypes.

**Implications of all the available evidence**

Serum levels of a limited number of proteins can accurately predict eosinophilic and neutrophilic asthma and help characterise and quantify the contribution of different molecular pathways in the development of the major asthma phenotypes.

## Introduction

Asthma is a heterogeneous airway disease that presents with different types and degrees of inflammation often in association with airway wall structural changes. Infiltrating inflammatory granulocytes such as eosinophils and/or neutrophils and resident airway structural cells participate in the inflammatory and remodelling processes of asthma through distinct mechanisms.^1^

Type-2 (T2) inflammation is characterized by the increased expression of T2-associated cytokines such as IL-4, IL-5, and IL-13 in the bronchial mucosa.^2^ Severe eosinophilic asthma is generally associated with late-onset disease and such patients present with frequent exacerbations, nasal polyps, fixed airflow obstruction, and dependence on oral corticosteroid therapy, and are responsive to T2-directed biologic therapies such as anti-IL5, anti-IL5Rα or anti-IL4Rα antibody treatments.^3^^,^^4^ Sputum eosinophil counts may reflect more reliably the eosinophilic inflammation of the airway than a blood eosinophil count.^5^ However, sputum induction is invasive and is not always successful in producing adequate samples from patients with asthma.^6^, ^7^, ^8^ Blood eosinophil counts are used as a proxy for sputum eosinophilia with a moderate to good correlation of blood eosinophil count with sputum eosinophil counts.^9^^,^^10^ Raised blood eosinophil counts in asthma subjects are associated with higher bronchial hyperresponsiveness, poor lung function, and higher serum IgE levels.^11^^,^^12^ In addition, patients with asthma with high blood eosinophil counts had more nasal polyps, a greater decline in lung function, higher levels of serum IgE and of fractional exhaled nitric oxide (FeNO), all biomarkers of T2 inflammation.^13^ In clinical practice, serum IgE levels are used to identify the allergic asthma phenotype whilst current or historical blood eosinophil counts above 150–300/μL and FeNO levels of ≥25 ppb are used to indicate eosinophilic or T2 asthma.^14^, ^15^, ^16^

Neutrophilic asthma, which is considered as a non-T2 or T2-low asthma, has been defined by a raised neutrophil count in sputum samples without raised eosinophil counts and has been linked to the presence of activated Type 1 and Th17 cells, along with an over-expression of IFN-γ, TNFα, IL-1β, and IL-17.^17^ Clinically, neutrophilic asthma is characterised by less frequent exacerbations than eosinophilic asthma with a moderate degree of airflow obstruction.^17^ A study of neutrophilic asthma defined by raised blood neutrophil count with low blood eosinophil counts, found that this was associated with increased levels of C-reactive protein, matrix metalloproteinase-9, IL-6, leptin, and soluble urokinase plasminogen activator receptor serum levels.^13^ This non-T2 or T2-low neutrophilic phenotype does not have any targeted biologic therapies since the key driving pathways are unclear.^18^ There is a need for a more granular definition of asthma phenotypes using blood biomarkers not only to inform the underlying molecular pathways but also to facilitate personalised therapeutic choices. We hypothesise, therefore, that large-scale proteomic analysis of serum proteins in well-characterised patients will identify markers which may be useful in determining subtypes of asthma.

In the present study, we used high-resolution proteomic profiles in 686 asthma participants with mild-moderate and severe disease from two cohorts to identify a set of serum proteins that would be predictive of either eosinophilic or neutrophilic asthma status as defined by the established clinical thresholds of granulocyte counts in both blood and sputum and investigate the underlying molecular pathways.^11^^,^^13^ We compared the serum biomarkers identified and their predictive ability to that of sputum biomarkers obtained in a subset who provided both blood and sputum samples.

## Methods

### Study population and data collection

We combined patients with mild-moderate and severe asthma from the Unbiased Biomarkers for the Prediction of Respiratory Disease Outcomes (U-BIOPRED) and the Airways Disease Endotyping for Personalized Therapeutics (ADEPT) cohorts to increase the contrast between severe and non-severe asthma and have a wider spectrum for severe asthma as the UBIOPRED cohort is composed mainly of patients with severe asthma while ADEPT is mainly mild-moderate.^19^^,^^20^ U-BIOPRED is a European multicenter cross-sectional study of asthmatic participants from 12 European countries and ADEPT is a European and North American study with asthmatic participants.

In both cohorts, serum (n = 574) and sputum (n = 182) samples were collected from participants along with demographic, clinical, and physiologic data. For (n = 62) ADEPT participants, an additional serum sample was collected two weeks after recruitment. Sputum samples were obtained via inhalation of hypertonic saline solution and sputum plugs were subsequently analysed as described elsewhere^17^^,^^19^ with eosinophil and neutrophil counts measured as markers of inflammation. Sputum supernatants were stored for later proteomic assay. Serum samples were also stored at the same time and blood neutrophil and eosinophil counts were obtained via routine hospital tests. Missing demographic and clinical data (missingness rates in Supplementary Table S1) was imputed using k nearest neighbours (number of nearest neighbours being 5), using the r package, VIM or number imputed.^21^

A total of 1129 proteins were originally measured from sputum supernatants and serum samples using the SOMAscan proteomic assay.^22^ After excluding proteins that did not fall within the validity range provided by Somalogic, 1129 serum proteins and 1125 sputum proteins were available in UBIOPRED samples and 1129 serum and 1128 sputum proteins were in ADEPT samples. Only considering proteins that were measured in both studies and both media, our final data included 1124 proteins measured in 574 patients with serum proteomic profile and 182 patients with sputum proteomic profile – some patients having both sputum and serum profiles (n = 178, 70 from UBIOPRED and 108 from ADEPT).

The data was firstly normalised to remove hybridization variation within a run, followed by median normalization to remove other assay biases. The data was z-score standardised and subsequently log-transformed and centred.^23^

The blood eosinophilic status (high vs. low) of participants was determined using a cut-off of blood eosinophil count of ≥300/*μ*L.^6^ Due to the lack of any agreed definition of neutrophilic asthma based on blood neutrophilic count, we used a cut-off of ≥7500/*μ*L, being the upper range of the normal limit of the blood neutrophil count.

Sputum eosinophil and neutrophil status was defined using the proportion of each cell type among granulocytes: high sputum eosinophil and neutrophil status were defined as ≥1.5% and ≥73.6% of the sputum granulocytic count, respectively.^7^

Because alternative cut-off points have been used regarding the definition of eosinophilic and neutrophilic asthma and to interrogate the validity and/or specificity of our findings, we have considered alternative cut-offs for eosinophilic and neutrophilic status in both serum and sputum samples: ≥3% and ≥150/μL for sputum and blood eosinophilic status respectively, and ≥60% and 5000/μL for sputum and blood neutrophilic status respectively.^12^^,^^24^^,^^25^

### Ethics

Ethics approval was obtained at all the participating centres for both studies and all participants gave written informed consent (ClinicalTrials.gov identifier: NCT01982162 & NCT01274507). Inclusion, ethical committees and initial stratification are detailed elsewhere.^19^^,^^20^

### Statistical analyses

#### Variable selection

We used least absolute shrinkage and selection operator (LASSO)-penalised logistic regression in a stability selection framework to identify a sparse set of proteins explaining eosinophilic or neutrophilic (binary) status separately from both serum and sputum proteomic profiles.^26^ For each outcome (eosinophilic or neutrophilic status) and each biosample (serum or sputum) separately, the penalised regression model was run for 1000 iterations on 50% subsamples of the study population. For a given value of the penalty we calculated the per-protein selection proportion as the number of times across the 1000 subsamples that specific protein was selected in the model. The calibration of the penalty (controlling the sparsity of the LASSO model) and of the selection proportion above which a feature is considered as being stably selected (controlling the stability of the model, conditional on the sparsity parameter) is undertaken by maximising a score measuring a negative log-likelihood under the hypothesis of equiprobability of selection (i.e. instability). These analyses were conducted using the “sharp” R package.^26^

Our variable selection models were adjusted for age and sex only and subsequently (by not penalising corresponding variables) for established clinical variables including body mass index (BMI), age of onset of asthma, asthma control questionnaire (ACQ) score, predicted forced expiratory volume (FEV1), presence or absence of nasal polyps, oral corticosteroid dose, smoking history in pack years, number of exacerbations per year and FEV1/forced vital capacity (FVC) ratio, as listed in Table 1.^27^

**Table 1:** Characteristics of asthma participants who contributed blood and sputum samples.

	Sputum				Blood			
	Total	ADEPT	UBIOPRED	p-value	Total	ADEPT	UBIOPRED	p-value
n participants at baseline	182	109	73	–	574	108	466	–
n participants with serum and sputum samples at baseline (%)	178 (97.8%)	108 (99.1%)	70 (95.9%)	–	178 (31.0%)	108 (100%)	70 (15.0%)	–
Age onset (mean (sd))	24.49 (17.53)	23.13 (16.05)	26.54 (19.50)	0.201	24.93 (17.91)	23.25 (16.08)	25.33 (18.32)	0.277
FEV_1 (%predicted)_ (mean (sd))	73.21 (20.20)	76.37 (18.20)	68.04 (22.30)	0.008	71.72 (21.89)	76.44 (18.27)	70.57 (22.56)	0.013
FEV_1/_FVC (mean (sd))	0.64 (0.12)	0.66 (0.11)	0.6 (0.13)	0.001	0.64 (0.13)	0.66 (0.11)	0.64 (0.13)	0.077
ACQ5 (mean (sd))	1.56 (1.10)	1.28 (0.86)	2.00 (1.30)	<0.001	1.87 (1.20)	1.28 (0.86)	2.01 (1.23)	<0.001
BMI (mean (sd))	26.83 (4.58)	26.24 (3.81)	27.71 (5.45)	0.034	28.04 (5.78)	26.23 (3.82)	28.47 (6.07)	<0.001
Pack years of smoking (mean (sd))	4.70 (10.10)	1.54 (2.94)	15.45 (16.61)	<0.001	9.60 (15.04)	1.55 (2.95)	15.17 (17.36)	<0.001
Exacerbations/year	1.23 (1.89)	0.28 (0.81)	1.99 (2.15)	<0.001	1.84 (2.14)	0.21 (0.65)	2.04 (2.17)	<0.001
Nasal polyps	43 (23.6)	21 (19.3)	22 (30.1)	0.130	156 (27.2)	21 (19.4)	135 (29.0)	0.059
OCS dose (mean (sd))	2.67 (6.10)	0.07 (0.72)	11.82 (7.72)	<0.001	10.69 (12.06)	0.07 (0.72)	16.60 (11.33)	<0.001
Age (years)	45.79 (13.92)	42.79 (13.83)	50.26 (12.89)	<0.001	48.72 (14.56)	42.87 (13.86)	50.08 (14.40)	<0.001
Sex. Male n (%)	79 (43.4)	47 (43.1)	32 (43.8)	1.000	237 (41.3)	46 (42.6)	191 (41.0)	0.844
Sex. Female n (%)	103 (13.22)	62 (56.9)	41 (56.2)		337 (58.7)	62 (57.4)	275 (59.0)	
Sputum neutrophil proportion (mean^a^ (sd))	56.46 (17.63)	59.42 (8.26)	52.05 (25.42)	0.005	55.40 (21.62)	59.46 (8.29)	52.54 (27.05)	0.011
Sputum eosinophil proportion (mean^b^ (sd))	7.52 (13.22)	3.97 (2.87)	12.83 (19.48)	<0.001	8.67 (14.73)	3.94 (2.87)	12.02 (18.38)	<0.001
Blood neutrophils (10^3^/μL) (mean (sd))	3.96 (2.47)	3.21 (1.96)	5.96 (2.59)	<0.001	4.78 (2.73)	3.23 (1.95)	5.40 (2.75)	<0.001
Blood eosinophils (10^3^/μL) (mean (sd))	0.43 (0.71)	0.49 (0.82)	0.28 (0.22)	0.119	0.36 (0.50)	0.49 (0.82)	0.31 (0.27)	0.002
Severity (%)	98 (53.8)	40 (36.7)	58 (79.5)	<0.001	420 (73.2)	40 (37.0)	380 (81.5)	<0.001

Numbers are presented for the full study population and stratified by study.

ACQ5: Asthma Control Questionnaire with 5 questions; BMI: Body Mass index; FEV1: Forced expiratory volume in 1 s; FVC: Forced vital capacity; MMA Mild-moderate asthma; OCS: Oral corticosteroid; SD: Standard deviation. Data shown as mean (SD), unless otherwise stated.

a Percentage of granulocytes which are neutrophils (asymmetrical standard deviations).

b Percentage of granulocytes which are eosinophils (asymmetrical standard deviations).

To assess the reproducibility of our main variable selection model (stability selection logistic LASSO), we re-ran the stability selection LASSO on 100 independent subsamples composed of 80% of the data. We report the resulting 100 per-protein selection proportion obtained across the (N = 100) subsamples.

#### Model performance assessment

We quantified the predictive abilities of the selected proteins by running series of unpenalised logistic regression models. The model coefficients were estimated through 1000 iterations of the train-test split procedure where the datasets were randomly split into 80% training and 20% testing subsets over the entire dataset. Each model was refitted on the training set and then subsequently tested on the corresponding 20% of un-seen observations to report area under the curve (AUC) with 95% confidence intervals (CIs). For further evaluation, the recalibrated model was tested on the repeated measurements from participants of the ADEPT study. As no patients with high neutrophil count were followed-up in the ADEPT cohort, that additional validation was restricted to serum eosinophilic status.

To assess the amount of information that the protein levels bring over and above that of established clinical factors listed above, we ran a series of logistic models including these factors and sequentially adding stably selected proteins (from the model adjusted for clinical factors) in decreasing order of selection proportions and report the corresponding AUC (and 95% CI) in the test set.

### Role of funders

The funders had no role in the design and conduct of the study; collection, management, analysis, and interpretation of the data; and preparation, review, or approval of this manuscript.

## Results

### Study population overview

Summary statistics of the key demographic and clinical characteristics of participants are reported in Table 1 for the full study population and the two contributing cohorts separately. Our study included slightly more women than men (56.6%) and the average age at onset was 24.5 years. Both characteristics were similar in both studies. By design, the ADEPT study included milder asthma cases compared to UBIOPRED participants, resulting in UBIOPRED participants showing (i) reduced lung function, (ii) higher BMI, (iii) higher exposure to tobacco smoking, (iv) higher sputum eosinophil and serum neutrophil levels, (vi) lower sputum neutrophil levels, and (vi) higher use of oral corticosteroids (OCS).

### Serum and sputum proteins associated with eosinophilic status

**Fig. 1:**
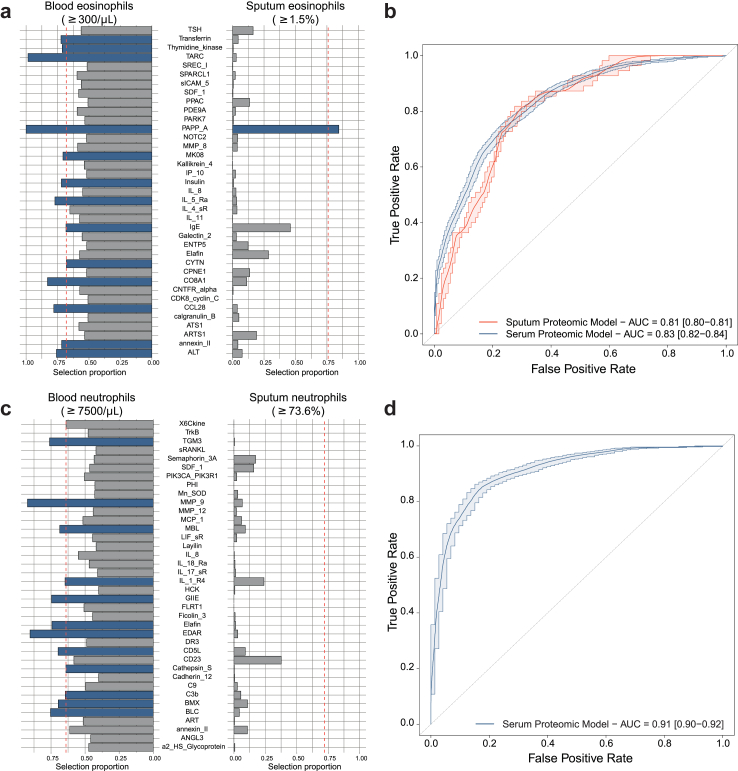
**Proteomic profiling of blood phenotypes from serum and sputum proteomics.** We determined whether blood eosinophil (a, b) and neutrophil (c, d) status was predictive using a logistic LASSO stability selection model to identify a sparse set of proteins. We report the per-feature selection proportion calculated across the N = 1000 subsamples for the N = 37/38 proteins with the highest selection proportions in blood (left panel) and sputum (right panel) levels. The vertical dashed line represents the calibrated selection proportion threshold for stably selected proteins. Receiver Operating Characteristics analyses were conducted by fitting a logistic regression model with selected variables and predicting eosinophilic (b) and neutrophilic (d) asthma status in the test dataset using blood or sputum data.

A total of 13 serum proteins were stably selected (with selection proportion greater than the 66% calibrated threshold) in the logistic LASSO stability selection analyses of serum eosinophilic status (Fig. 1a). These included Pregnancy-associated plasma protein A (PAPP-A) and thymus- and activation-regulated chemokine (TARC, now known as CC chemokine ligand 17 (CCL17)) with selection proportions of 1 and 0.98 respectively. Running the stability selection LASSO on 100 different partitions of the study population, we found that PAPP-A and TARC/CCL17 were the two most consistently selected proteins (Supplementary Fig. S1). The inclusion of all 13 stable proteins in the logistic regression model predicting serum status yielded an AUC of 0.83 (95% CI 0.82–0.84 (Fig. 1b).

**Fig. 2:**
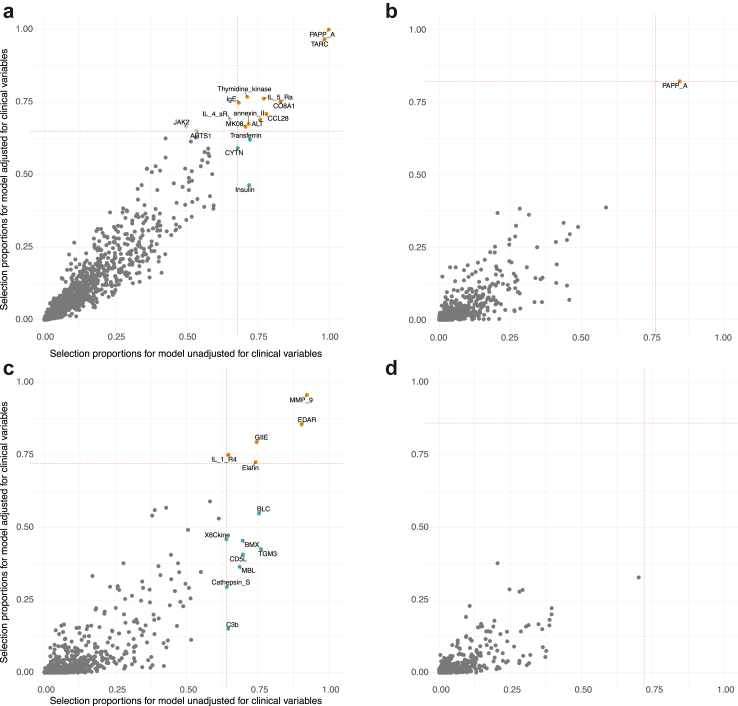
**Comparison of proteins selected in models adjusted and unadjusted for clinical variables for serum eosinophilic asthma status (a) and sputum eosinophilic asthma status (b), serum neutrophilic asthma status (c), and sputum neutrophilic asthma status (d)**. We compared the per-feature selection proportion calculated across the N = 1000 subsamples for adjusted and unadjusted models. Dashed lines represent threshold for stably selected proteins for models. Grey points represent proteins not selected in each model, orange points are those selected in both models, blue represents those selected in the unadjusted model and green points are proteins selected in the adjusted model.

**Fig. 3:**
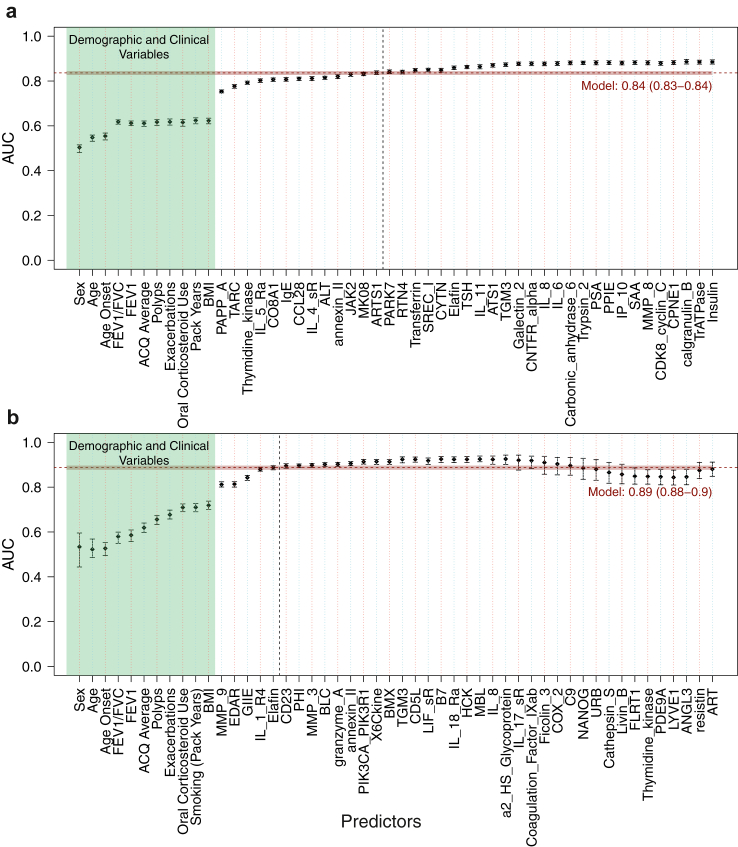
**Predictive performance of serum proteins beyond established clinical factors.** Results from a series of logistic regression models for eosinophilic (a) and neutrophilic (b) status including established clinical factors and sequentially adding stably selected proteins in descending order of selection proportion from stability selection LASSO model adjusted for clinical factors. Model coefficients recalibrated on 1000 80% training sets show the mean area under the curve (AUC) (and 95% CI) obtained in the 20% testing sets. The variables on the left of vertical dashed black line are those with selection proportions greater than the calibrated threshold and represent stably selected proteins. The model adjusted for clinical factors included 13 stably selected serum proteins for eosinophilic status (a) and 5 serum proteins for neutrophilic status (b). Corresponding AUC (horizontal dashed line) and 95% CI (bold region) are also represented.

Stability selection models adjusted for defined clinical factors selected 13 proteins including PAPP-A and TARC/CCL17 along with eight other proteins that were also selected in the unadjusted model (Fig. 2a), in addition to three proteins: Janus kinase 2 (JAK2), Interleukin 4 soluble receptor (IL-4sR), and Endoplasmic reticulum aminopeptidase 1 (ARTS1) yielding an AUC of 0.84 (95% CI 0.83–0.84). Models including clinical variables and sequentially adding proteins in descending order of selection proportion (Fig. 3a) showed that the model including only PAPP-A yielded an AUC of 0.75 (95% CI: 0.75–0.76) and that the addition of TARC/CCL17, an AUC of 0.78 (95% CI: 0.77–0.78). This was higher than that of the model that only included established clinical markers [AUC = 0.62 (95% CI: 0.61–0.63).

The proteomic model for eosinophilic serum status was fitted to data collected from the ADEPT cohort fourteen days after enrolment (Supplementary Table S2) and yielded an AUC of 0.76 (95% CI 0.63–0.89), hence supporting the generalisability of our results.

When predicting sputum eosinophil status using sputum proteomic data, only PAPP-A was stably selected (Fig. 1a) yielding an AUC of 0.81 (95% CI 0.80–0.81) (Fig. 1b). PAPP-A was also selected in the model adjusting for established clinical variables (Fig. 2b), suggesting that it provided relevant information on eosinophilic status that was not captured by the clinical variables: the AUC of the adjusted model including PAPP-A was at 0.79 (95% CI 0.76–0.82), compared to 0.63 (95% CI 0.58–0.67) for the model only including established clinical factors (Supplementary Fig. S2).

### Serum and sputum proteins associated with neutrophilic status

Stability logistic LASSO (Fig. 1c) for neutrophilic status resulted in 12 stable serum proteins (with selection proportion above calibrated threshold of 0.64) with a corresponding AUC of 0.91 (95% CI 0.90–0.92) (Fig. 1d). The proteins with the highest selection proportions were matrix metalloproteinase (MMP-9) (selection proportion: 0.85) and ectodysplasin A receptor (EDAR) (selection proportion: 0.80). Running the stability LASSO (Supplementary Fig. S3) models on 100 different subsets of the study population, we found that MMP-9 and B lymphocyte chemoattractant (BLC) were the most frequently selected proteins.

While adjusting for clinical variables, 5 proteins (MMP 9, EDAR, Phospholipase A2 Group IIE (PLA2G2E), Interleukin 1 Receptor Type 4 (IL-1-R4) now known as IL1RL1/IL33R and Elafin) were stably selected, which were all selected in the unadjusted model (Fig. 2c). These additional proteins increased the AUC from 0.72 (CI: 0.70–0.74) (for the model including just clinical covariates) to 0.89 (CI: 0.88–0.90) with the addition of proteins (Fig. 3b).

Irrespective of the adjustment for established clinical factors, no sputum proteins were associated with neutrophilic status using logistic LASSO stability analysis (Fig. 1, Fig. 2d).

### Using alternative cut-off points for defining eosinophilic and neutrophilic asthma

Changing the eosinophil serum cut-off for classification from 300/μL to a lower threshold of 150/μL, PAPP-A and IgE were consistently chosen (Supplementary Fig. S4). IL5Rα and TARC/CCL17 which were prominent in the original analysis were no longer selected with this alternative definition.

The cut-off for classifying eosinophil high subjects using sputum data for the original analysis was ≥1.5%, while other studies have used a 3%. Using that alternative definition of eosinophil high status (Supplementary Fig. S5), our logistic LASSO model selected PAPP-A and Cathepsin G (which was not selected in our original analysis) as being associated with eosinophilic status.

Although neutrophilic asthma does not have an agreed definition, an alternative threshold of 5000/μL was used. MMP-9, Cathepsin S, and GIIE/PLA2G2E (Supplementary Fig. S6) overlapped with those found in the original definition. Finally, changing the cut-off for sputum neutrophil classification from 73.6% to 60% led to 3 sputum proteins being stably selected namely Carbonic anhydrase I, CD23, and CD27 (Supplementary Fig. S7).

## Discussion

We used the U-BIOPRED and ADEPT asthma datasets to provide serum and sputum proteomic biomarkers linked to both blood and sputum eosinophil and neutrophil cut-off levels in particularly patients with severe asthma, as these represent markers of T2 and non-T2 inflammation, respectively. The models used proved to be successful with AUCs ranging from 0.80 to 0.91 showing the class separation capability of the serum and sputum proteomic data. The stably selected proteins we identified add the extra information and premise that clinical covariates alone cannot account for and could therefore provide more granular information on the biological pathways that are underlying these asthma phenotypes.

We identified a set of proteins that were associated with blood and sputum eosinophilia and of these, only PAPP-A was identified in both blood and sputum using the cut-off of ≥300/μL and ≥1.5%, respectively. PAPP-A was found to contribute information over and above that from age, sex, and established clinical factors and their association was found to be robust. An additional twelve serum proteins were associated with blood eosinophilic status, many of which have been previously linked to T2 inflammation as discussed below. Predictive performances for eosinophilic status were slightly better for models using serum proteomic data compared to models based on sputum data, although this could be due to the larger sample size of serum versus that of sputum. To verify this, the analysis was re-run on a (n = 182) subsample of the serum data, where the key characteristics of the participants in that subsample were controlled to be the same as in the full population. The AUCs for serum eosinophilia were reduced to 0.73 (95% CI: 0.72–0.74) and no protein was selected for neutrophilic asthma. This may suggest that part of the sputum data's lower predictive power/strength was related to the smaller sample size.

Many but not all of these serum proteins have already been associated with T2 eosinophilic inflammation. Serum PAPP-A, an insulin-like growth factor binding protein 4 protease (IGFBP-4), has been previously reported to be higher in the serum of patients with asthma compared to patients without asthma^28^^,^^29^ and in patients with severe allergic asthma treated with the anti-IgE monoclonal antibody omalizumab, PAPP-A serum levels were reduced.^29^ With the readjustment of the eosinophilic count threshold to blood eosinophil count of ≥150/μL and sputum eosinophil of ≥3%, PAPP-A and IgE, and PAPP-A and Cathepsin G were picked in blood and sputum, respectively. This reinforces the value of PAPP-A in both serum and sputum and its association with T2 inflammation, but its functional role and site of action remain to be confirmed in the airways.

The biomarkers IgE and IL5Rα that were picked up are the target of 2 different biological therapies, namely omalizumab that targets IgE, and the monoclonal antibody, benralizumab, that targets IL5Rα.^16^ Serum IgE levels have been used to determine the amount of omalizumab to be given to patients with severe allergic asthma for optimal response,^30^ while blood eosinophil counts have been used as a biomarker to choose those with the greatest likelihood of responding to benralizumab.^16^

Cystatin SN (CYTN) encoded by CST1 is a cysteine proteinase inhibitor that has been found to be higher in sputum supernatants and serum in poorly-controlled asthmatics compared to well-controlled asthma^31^ and is also upregulated in allergic rhinitis.^32^ Cystatin SN prevents the disruption of the airway epithelial barrier by allergen proteases.^31^ TARC/CCL17 and IgE levels are upregulated in elderly patients with asthma (and allergic asthma).^33^ TARC/CCL17 is a chemoattractant of Th2 cells,^34^ which explains its association with eosinophilic asthma but not the link with ageing which needs further research. Suppression of TARC/CCL17 could potentially reduce airway hyperresponsiveness and eosinophilia whilst another study found that TARC/CCL17 levels negatively correlated with FEV1%.^35^^,^^36^ Dupilumab has been shown to reduce serum TARC, alongside a reduction in plasma eotaxin-3 and serum periostin with serum IgE in severe eosinophilic asthma,^37^ supporting TARC/CCL17 as being a potential biomarker of eosinophilic asthma and of those responding to Dupilumab.

CCL28 is expressed by mucosal epithelial cells and attracts many immune cells including T-cells and eosinophils. Severe respiratory viral infection has been linked to the recruitment of IL-13–producing Th2 cells via induction of dendritic cell high-affinity IgE-receptor (FcεRI), and CCL28.^38^^,^^39^ CCL28 may also have a role in asthma pathogenesis with high levels of CCL28 in sputum and CCL28 receptors being expressed on eosinophils in patients with asthma and atopic disease.^40^, ^41^, ^42^, ^43^

For neutrophilic asthma, we identified 12 serum proteins at a blood neutrophil count cut-off of ≥7500/μL and of these, five (MMP-9, EDAR, GIIE/PLA2G2E, IL-1-R4/IL1RL1, and Elafin) added to the predictive power of established clinical factors. Of the 12 serum proteins, 5 were related to protease/anti-proteases: MMP-9, Elafin, BLC (CXCL13) and cathepsin S, 3 to other enzymes: BMX (non-receptor tyrosine kinase), TGM3 (Transglutaminase 3) and GIIE/PLA2G2E (secreted phospholipase A2), while others related to IL-1_R4/IL1RL1 (the IL33 receptor) and EDAR (Ectodysplasia A receptor). Of these, MMP-9, and Cathepsin S can be directly associated with neutrophils. MMP-9, which is produced mainly by macrophages and neutrophils, is involved in airway and lung remodelling, with elevated levels reported in blood, sputum, and bronchoalveolar lavage fluid of patients with asthma.^44^, ^45^, ^46^ Cathepsin S which is a lysosomal cysteine protease plays an important role in the regulation of oxidant-induced airway hyperresponsiveness and neutrophil recruitment in mice^47^ and increased levels have been shown in allergic lung inflammation with a role in lung remodelling being proposed.^48^^,^^49^ BCL (CXCL13) is a potent B-cell chemoattractant factor that interacts with the CXCR5 receptor expressed on B cells.^50^ CXCL13 levels in the asthmatic children's sputum were significantly higher than those in control group^51^ and CXCL13 levels in plasma or serum of adults with asthma were increased in patients with an exacerbation history or during exacerbations.^52^^,^^53^ The secreted phospholipase A2 (sPLA2) activated during IgE-mediated activation of mast cells leading to the generation of eicosanoids has been shown to be of increased activity in bronchoalveolar lavage fluid of patients with asthma, particularly after allergen challenge.^54^^,^^55^ sPLA2 can promote the recruitment of neutrophils.^56^ Interestingly, activation of the IL-33 receptor on mast cells by the alarmin IL-33 can lead to neutrophilic inflammation.^57^ When the blood neutrophil count threshold was reduced to ≥ 5000/μL, MMP-9, Cathepsin S, and sPLA2 remained associated with neutrophilic status.

No sputum proteins were associated with neutrophil status using a neutrophil count of ≥73.6%. With a lower neutrophil count cut-off of ≥60%, three proteins, Carbonic anhydrase 1, CD23 and CD27, were chosen. The role of these proteins in the context of neutrophilic asthma remains unclear although CD23 is the low-affinity IgE receptor, and CD27 is a member of the TNF receptor superfamily and interaction with CD70 is involved in the differentiation of B cells into plasma cells.^58^

There are some limitations to our study. Pooling the dataset from the U-BIOPRED and ADEPT could potentially introduce some bias especially in the analysis of blood phenotypes where >80% of the participants came for U-BIOPRED. The analysis of sputum phenotypes relied on more balanced contribution of both studies. Nevertheless, we could replicate results in the longitudinal measurements in ADEPT. In addition, our finding of fewer explanatory proteins in sputum data may be related to reduced statistical power for these analyses relying on a smaller number of samples available. In keeping, when the serum data was subsampled to match the sample size of the sputum analyses, fewer variables were selected, and the AUC was lower. However, the difference in sample size between sputum and serum does represent what is available in the real world as blood is easier to collect than sputum. Another limitation of this study is the assumption that a patient has either a high eosinophil or neutrophil status, hence overlooking participants with elevated eosinophils and neutrophils (“mixed” status). Future work should investigate asthma statuses jointly. Our sensitivity analyses used alternative (lower) cut-off values for blood cell counts which in turn may have diluted the effect of some proteins. That dilution effect may, at least, partially explain that 11 and 9 proteins found jointly associated with serum eosinophilic and neutrophilic status, respectively in our original analysis were not selected while considering these alternative thresholds. By increasing the number of participants with high granulocyte counts, the use of these alternative thresholds may have also increased the statistical power of our study, which may explain why our model selected with these revised thresholds (i) 14 and 13 proteins that were not selected in our original analysis of serum eosinophilic and neutrophilic status, respectively and, (ii) one additional protein (Cathepsin G) associated with sputum eosinophilic status and three proteins (CD27, Carbonic anhydrase and CD23), jointly associated with sputum neutrophilic status. Variations in the selection of variables for the different thresholds may also suggest competing proteins or distinct pathways relevant to lower eosinophilic asthma, prompting further investigation to explore which of these proteins are related to common pathways underlying serum and/or sputum eosinophilia and neutrophila and which may be related to the severity of these traits.

Nevertheless, our sensitivity analyses indicated that PAPP-A (in sputum and serum), and IgE (in serum only) were associated with eosinophilia irrespective of the cut-off used and that MMP 9, Cathepsin S, and BLC were consistently associated with serum neutrophilia. Overall, this lends plausibility to the underlying pathways these may contribute to.

In conclusion, targeted serum proteomic profiling could be used to characterise eosinophilic and neutrophilic phenotypes of patients with asthma and to obtain a more granular definition of these endotypes by evaluating and quantifying the contribution of some (non-)established perturbed pathways contributing to the asthma phenotypes. Due to very different numbers of serum and sputum samples available, their predictive performances cannot be compared directly, however, the serum model based on a subsample of the data showed reasonable performances and that selected proteins added clinically relevant information over that from established clinico–physiological parameters. Serum proteomic profiling could therefore be considered as a non-invasive and cost-effective alternative to sputum profiling for asthmatic phenotypic characterisation. However, because blood-based proteomic profiles may be affected by different organs, the proteins we identify may reflect other (and potentially distant) physiological processes. While our results remain valid in terms of patient stratification, inferred pathways should be carefully assessed on a patient-by-patient basis in a personalised treatment setting.

## Contributors

DV and MC-H are joint last authors. MC-H, KFC, DV, and IMA conceived the study and the analytical plan. KA, MC-H, BB, and DV performed the statistical analyses. NZK, KFC, PKB, S-ED, RD and IMA provided insights into the study design and results interpretation. KFC, IMA, and MC-H obtained funding. The UBIOPRED consortium made significant contributions by providing valuable data, information and offering valuable advice. All authors revised the manuscript for important intellectual content and approved the final manuscript. KA, KFC, NZK, IMA, DV and MC-H had full access to the data and take responsibility for the integrity of the data and the accuracy of the data analysis and for the decision to submit for publication.

## Data sharing statement

The data that support the findings of this study are available upon request to UBIOPRED steering committee.

## Declaration of interests

Prof M Chadeau-Hyam holds shares in the O-SMOSE company; consulting activities conducted by the company are independent of the present work. Prof Adcock reports consulting fees from GSK, Sanofi, Chiesi and Kinaset; speaker fees from AZ, Sanofi, Eurodrug and Sunovion; travel support from AZ; grants from GSK, MRC, EPSRC, Sanofi and NIEHS, which were independent of the present work. Dr. Dahlén reports consulting fees from Affiboby, AZ, Cayman Chemicals, GSK and Regeneron, and speaker fees from AZ, GSK and Sanofi, outside the submitted work. Prof Chung has received speaker fees from Novartis, AZ and Merck; honoraria for participating in Advisory Board meetings of GSK, Novartis, Roche, Merck, Trevi, Rickett-Beckinson, Nocion and Shionogi; and has received grants from MRC, EPSRC and GSK. Prof Chung is a member of the Scientific Advisory Board of the Clean Breathing Institute supported by Haleon. Dr Djukanovic declares consulting fees from Synairgen plc and lecture fees from GSK, ZenasBio and Celltrion. He holds shares from Synairgen and is Chair of the European Respiratory Society's Clinical collaboration on severe asthma (SHARP). The other authors have no conflict of interest to disclose.
