# Supplementary material for: Proteomic signatures of eosinophilic and neutrophilic asthma from serum and sputum
Source: eBioMedicine. 2023 Dec 20;99:104936. doi: 10.1016/j.ebiom.2023.104936 (PMC10776923; doi:10.1016/j.ebiom.2023.104936)
Supplement: Supplementary Material 1 [file mmc1.pdf]

Supplementary Material for: Proteomic signatures of eosinophilic and neutrophilic asthma from serum and sputum

Khezia Asamoah[1], Kian Fan Chung[2,3,4], Nazanin Zounemat Kermani[2,3], Barbara Bodinier[1], Sven-Erik Dahlen[5,6], Ratko Djukanovic[7,8], Pankaj K Bhavsar[3] , Ian M Adcock[2,3], Dragana Vuckovic[1, *], Marc Chadeau-Hyam[1†*] on behalf of the U-BIOPRED Study Group

[1]MRC Centre for Environment and Health & Department of Epidemiology and Biostatistics, Faculty of Medicine, School of Public Health, Imperial College London; [2]Data Science Institute, Department of Computing, Imperial College London, United Kingdom; [3]National Heart and Lung Institute, Imperial College London, London, United Kingdom; [4]Royal Brompton and Harefield Hospital, London, United Kingdom; [5]Institute of Environmental Medicine and Department of Medicine Huddinge, Karolinska Institutet, Stockholm, Sweden; [6]Department of Respiratory Medicine, Karolinska University Hospital, Stockholm, Sweden; [7]Clinical and Experimental Sciences, Faculty of Medicine, University of Southampton, Southampton, United Kingdom; [8]National Institute for Health Research Southampton Biomedical Research Centre, Southampton, United Kingdom;

b

b

* Joint last authors

† Corresponding author: Marc Chadeau-Hyam, Department of Epidemiology and Biostatistics, School of Public Health, Imperial College London, St Mary's Hospital, Norfolk Place, W21PG, London

m.chadeau@imperial.ac.uk

Contents:

Supplementary Methods
Supplementary Table S1-S2
Supplementary figures S1-S7

Supplementary methods

Penalized regression models are established approaches accommodating data with less observations than measurements. Of these, the Least Absolute Shrinkage and Selection Operator (LASSO) uses an L-1 penalisation and achieves variable selection by shrinking the regression coefficient of uninformative features exactly to zero and therefore improves models interpretability [1]. Calibration of the LASSO requires the definition of the penalty parameter, which controls the sparsity of the model. Classical calibration procedures relies on cross-validation, where the data is split (possibly several times) into several folds which are sequentially combined to define multiple training and testing sets. In that framework, the optimal penalty parameter is the one yielding, across all train/test combinations the lowest error.

In order to improve the reproducibility of the result, the LASSO can be used in a stability selection framework [2]: the model is applied on multiple subsamples of population. Per-feature selection proportions are then calculated as the proportion of subsamples where the given predictors have been selected. Features with selection proportions above a given threshold are then considered as stably selected. As recently proposed [3], the penalty parameter (controlling the sparsity of the model) and threshold in selection proportion (controlling the stability of the model) are here jointly calibrated by maximising a consensus score based on a two-sample z-test where the null hypothesis is the equiprobability of selection for all features.

[1] Tibshirani R. Regression shrinkage and selection via the lasso. J R Stat Soc 1996;58:267–88.
[2] Meinshausen N, Bühlmann P. Stability selection. J R Stat Soc Series B Stat Methodol 2010;72:417–73.
[3] Bodinier B, Filippi S, Nøst TH, Chiquet J, Chadeau-Hyam M. Automated calibration for stability selection in penalised regression and graphical models. J R Stat Soc Ser C Appl Stat 2023. https://doi.org/10.1093/jrsssc/qlad058.

Supplementary Table S1: Missingness rates for clinical variables

**Variable**	**Total percentage missing data (%)**	**Percentage missing data in UBIOPRED (%)**	**Percentage missing data in ADEPT (%)**
age_onset	2.75	3.38	0
fev1	4.98	5.91	0.93
fev1fvc	1.20	1.27	0.93
acqaverage	2.58	3.16	0
polyps	0	0	0
exacerbations	8.93	0.21	47.22
ocs	48.11	59.07	0
pack_years	54.30	66.67	0
bmi	0	0	0
demographic_data_sex	0	0	0
demographic_data_age	0	0	0

Supplementary Table S2. Area under the ROC (95% CI) for an unpenalized logistic regression model for eosinophilic status at 2 weeks, months in ADEPT participants of 13 stably-selected proteins for eosinophilic status

Timepoint	Total (n)	Eosinophil low (n)	Eosinophil high (n)	AUC (95% CI)
Day 14	62	43	19	0.76 (0.63, 0.89)

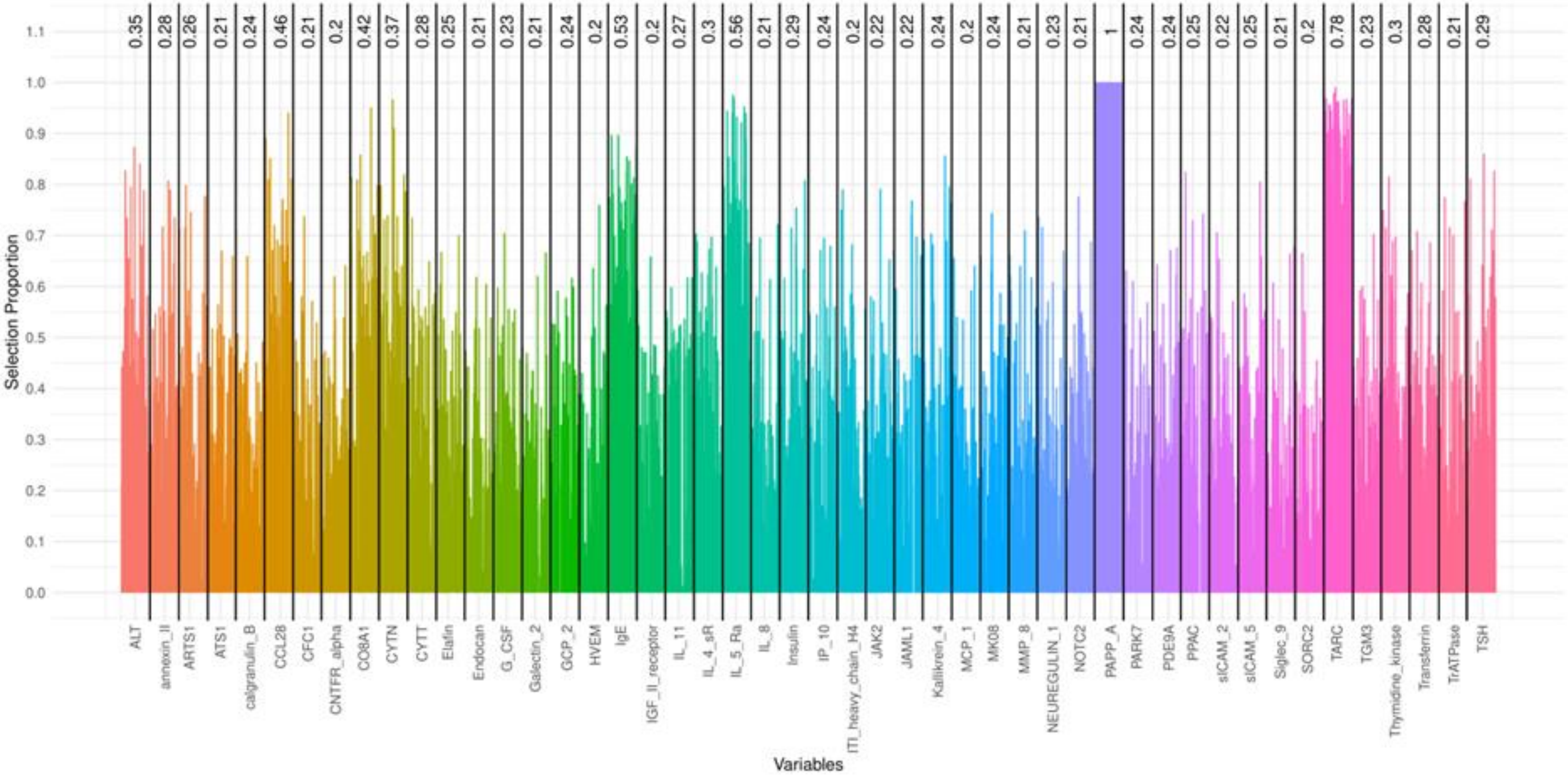

Supplementary Figure S1. Reproducibility of logistic LASSO stability results for serum eosinophilic asthma status. The stability selection algorithm was run on 100 different training sets, and we report the per-protein selection proportions we estimated across these 100 models and report for each protein the average selection proportion.

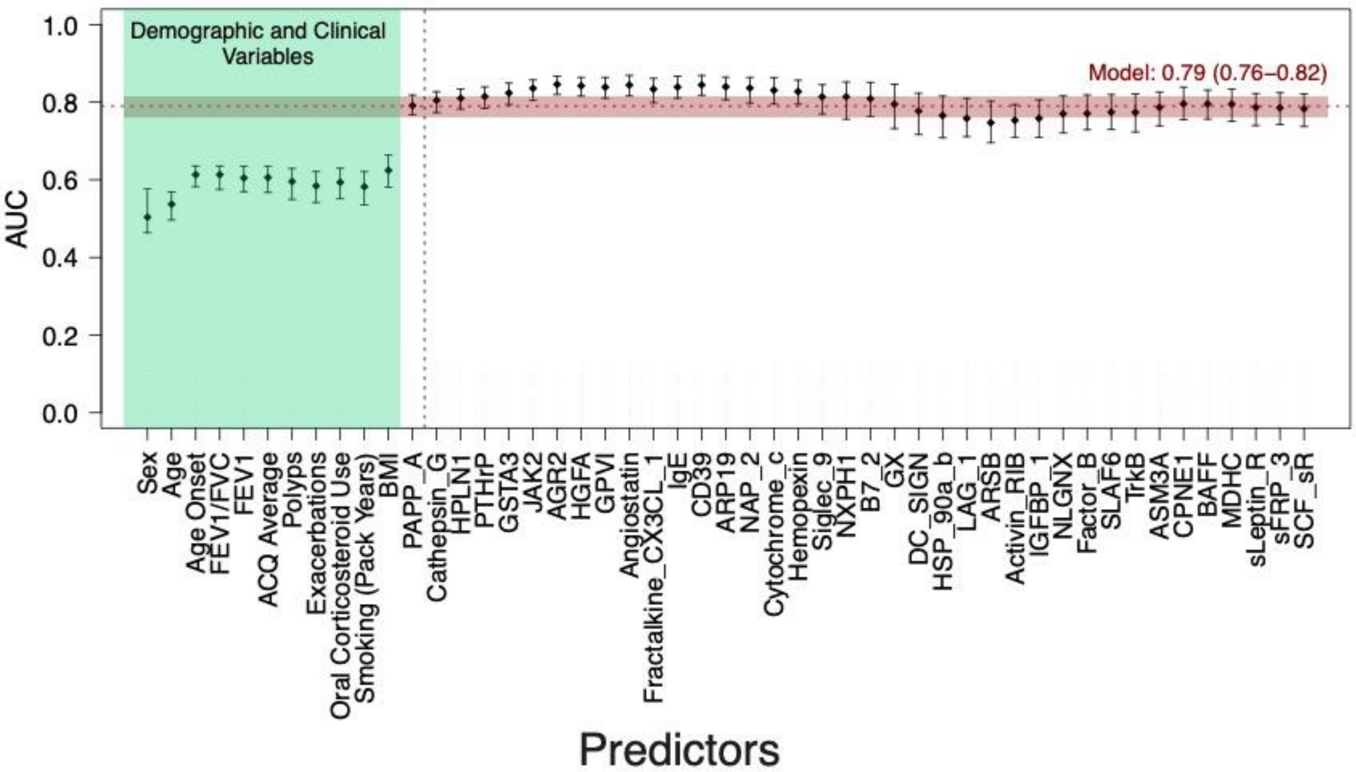

Supplementary Figure S2. Predictive performances of sputum proteins for eosinophilic asthma beyond established clinical factors. Results from a series of logistic regression models for eosinophilic status including established clinical factors and sequentially adding stably selected proteins in descending order of selection proportion form stability selection LASSO model adjusted for clinical factors. Models coefficients were recalibrated on 1,000 80% training sets and we report the mean AUC (and 95% CI) obtained in the 20% testing sets. The variables on the left of vertical dashed black line are those with selection proportions greater than the calibrated threshold and represent stably selected proteins. The model adjusted for clinical factors included a single stably selected sputum protein. Corresponding AUC (horizontal dashed line) and 95% CI (bold region) are also represented.

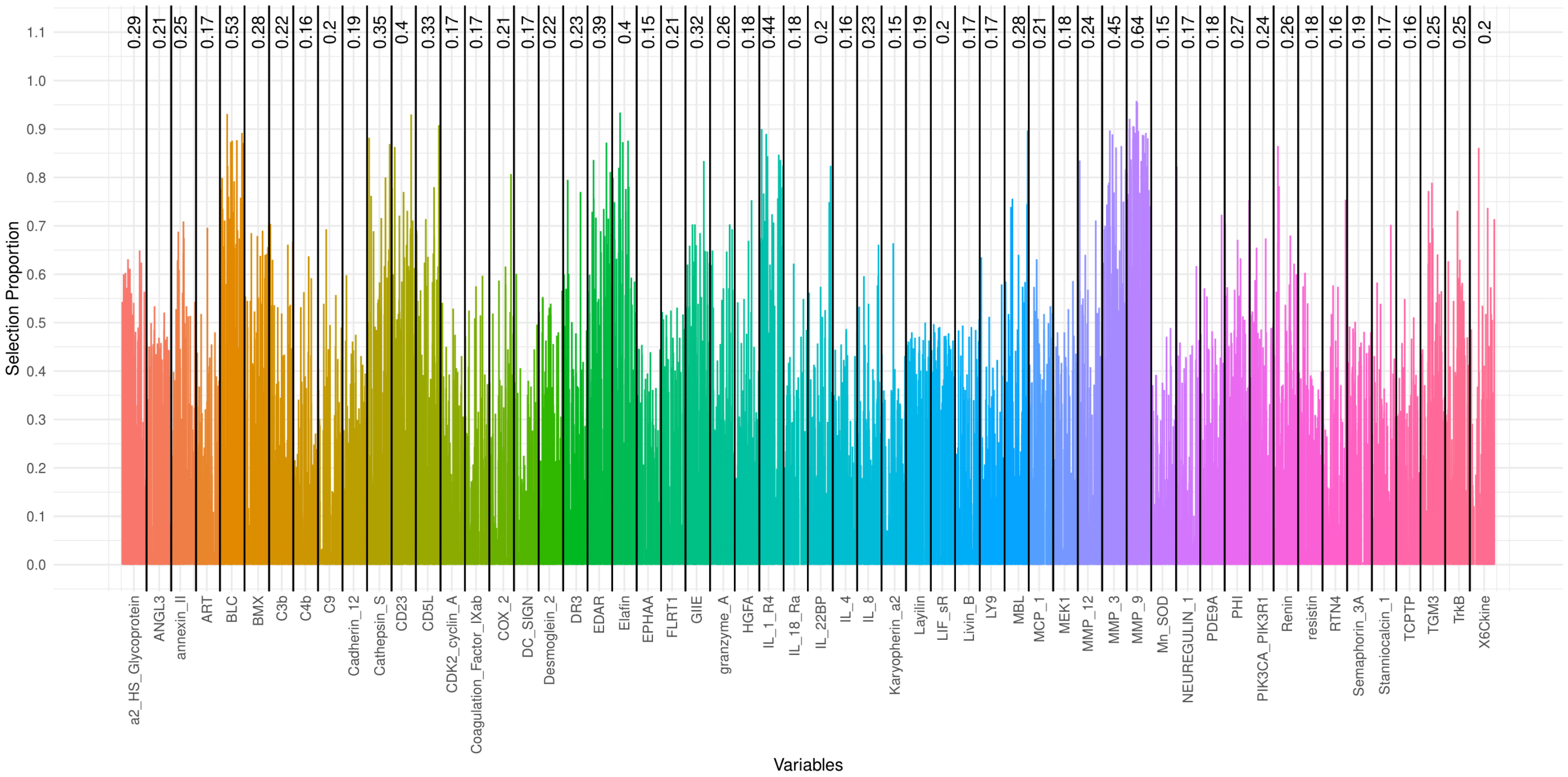

Supplementary Figure S3. Reproducibility of our logistic LASSO stability results for serum neutrophilic asthma status. The stability selection algorithm was run on 100 different training sets, and we report the per-protein selection proportions we estimated across these 100 models and report for each protein the average selection proportion.

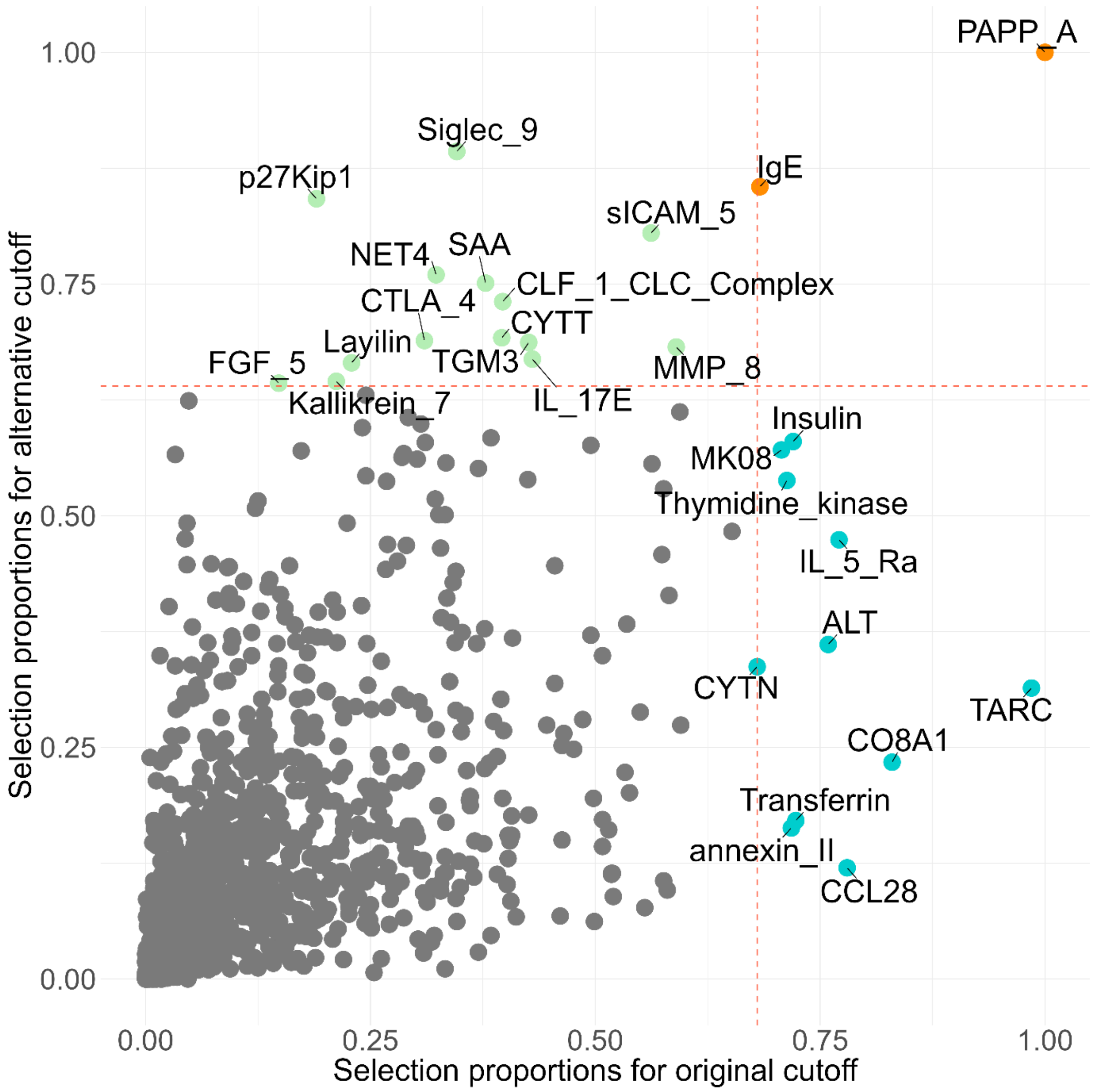

Supplementary Figure S4. Serum proteins associated with eosinophilic status: sensitivity to the definition of eosinophilic status. Per-protein selection proportion from the stability logistic LASSO for eosinophilic status using serum data. Selection proportions were estimated using 1,000 80% sub-samples of the study population. We considered patients with high Eosinophilic status if their serum eosinophil counts were greater than 300/µL (as in the main analyses) or, as a sensitivity analysis greater than 150/µL. Models were run for both definitions and thresholds in selection proportion, which were calibrated jointly with the penalty parameters, are represented as dashed horizontal. We report the per feature selection proportion and represent in blue the proteins found stably selected in the model for the original definition, in green for the model using the alternative definition of the eosinophilic status, and orange for features selected in both.

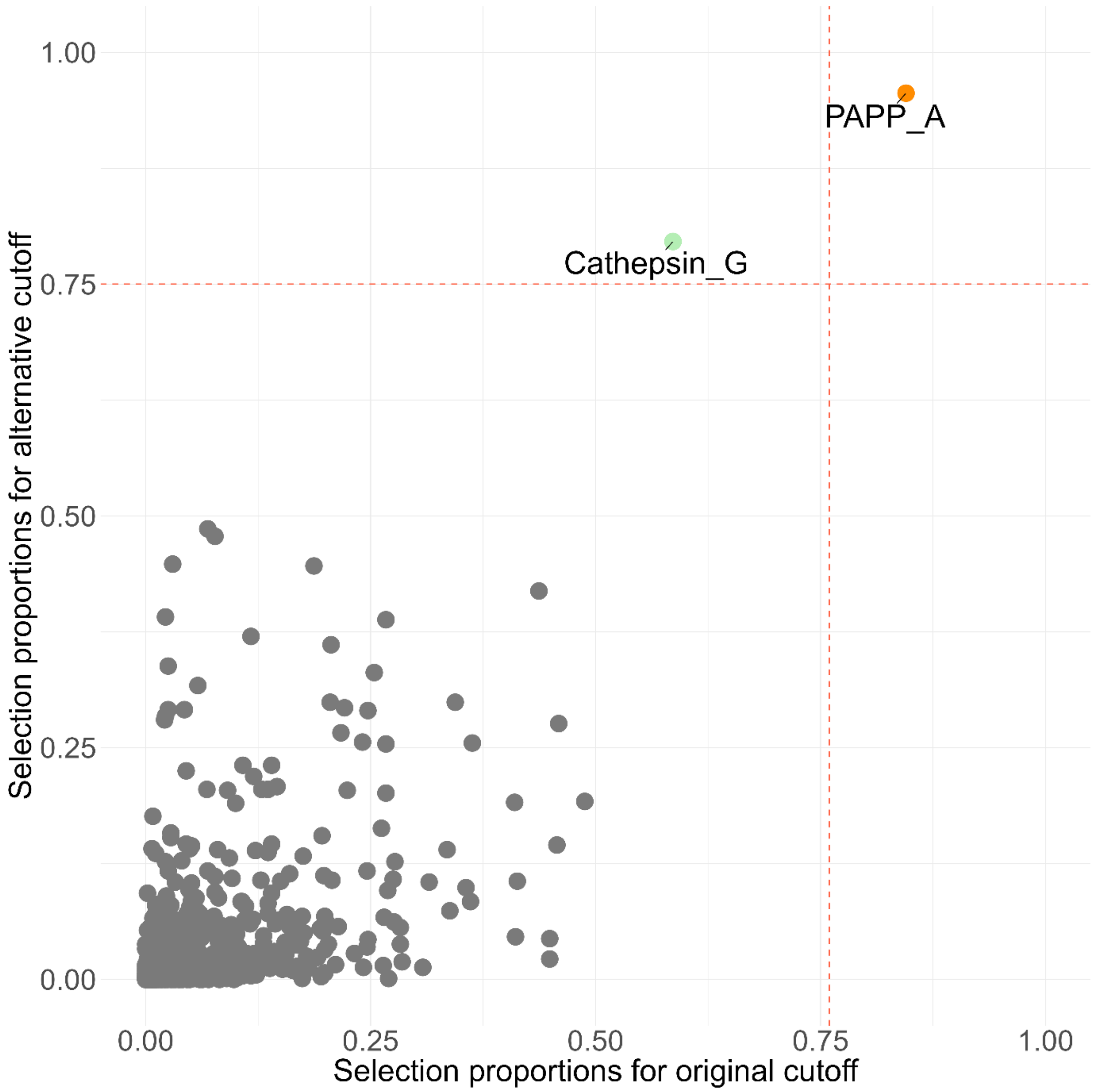

Supplementary Figure S5. Sputum proteins associated with eosinophilic status: sensitivity to the definition of eosinophilic status. Per-protein selection proportion from the stability logistic LASSO for eosinophilic status using sputum data. Selection proportions were estimated using 1,000 80% sub-samples of the study population. We considered patients with high Eosinophilic status if their sputum eosinophil proportion was greater than 1.5% (as in the main analyses) or, as a sensitivity analysis, greater than 3%. Models were run for both definitions and thresholds in selection proportion, which were calibrated jointly with the penalty parameters, are represented as dashed horizontal. We report the per feature selection proportion and in green features selected for the model using the alternative definition of the eosinophilic status, and orange for features selected in both.

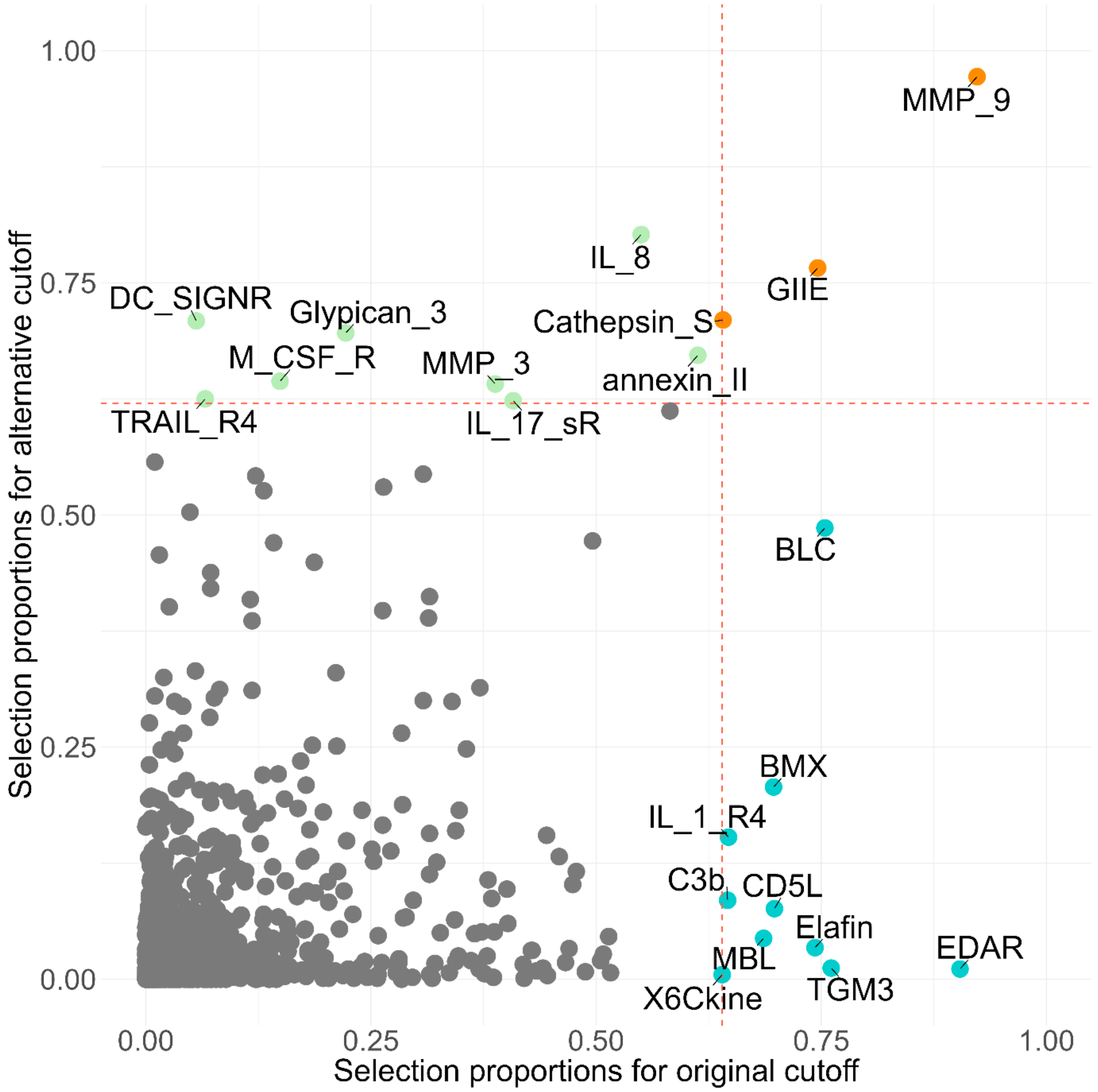

Supplementary Figure S6. Serum proteins associated with neutrophilic status: sensitivity to the definition of neutrophilic status. Per-protein selection proportion from the stability logistic LASSO for neutrophilic status using sputum data. Selection proportions were estimated using 1,000 80% sub-samples of the study population. We considered patients with high neutrophil status if their serum neutrophil counts were greater than 7500/µL (as in the main analyses) or, as a sensitivity analysis greater than 5000/µL. Models were run for both definitions and thresholds in selection proportion, which were calibrated jointly with the penalty parameters, are represented as dashed horizontal. We report the per feature selection proportion and represent in blue the proteins found stably selected in the model for the original definition, in green for the model using the alternative definition of the neutrophilic status, and orange for features selected in both.

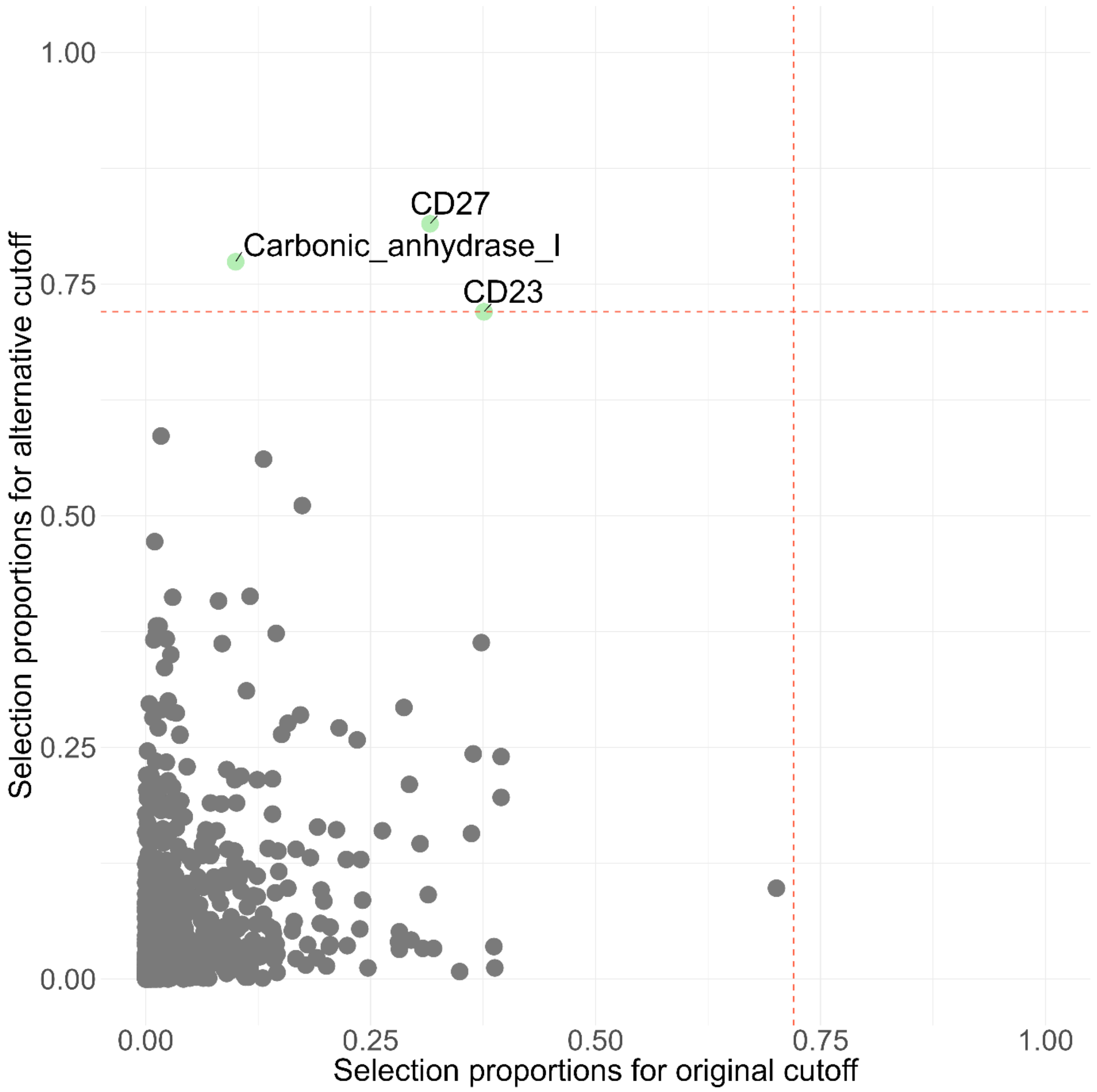

Supplementary Figure S7. Sputum proteins associated with neutrophilic status: sensitivity to the definition of neutrophilic status. Per-protein selection proportion from the stability logistic LASSO for neutrophilic status using sputum data. Selection proportions were estimated using 1,000 80% sub-samples of the study population. We considered patients with high neutrophilic status if their sputum neutrophil proportion was greater than 73.6% (as in the main analyses) or, as a sensitivity analysis greater than 60%. Models were run for both definitions and thresholds in selection proportion, which were calibrated jointly with the penalty parameters, are represented as dashed horizontal. We report the per feature selection proportion and represent in blue the proteins found stably selected in the model for the original definition, in green for the model using the alternative definition of the neutrophilic status, and orange for features selected in both.
